# Healthcare delivery for non-communicable diseases among breast cancer survivors in Sri Lanka: Is there a missed opportunity?

**DOI:** 10.3332/ecancer.2021.1301

**Published:** 2021-10-12

**Authors:** Don Thiwanka Wijeratne, Christopher M Booth, Sanjeewa Seneviratne, Bishal Gyawali, Matt Jalink, Malinthi Soysa, Sachith Abhayaratna, Hasitha Promod, Punika Wijesinghe, Sanjeeva Gunasekera

**Affiliations:** 1 Department of General Internal Medicine, Queen’s University, 94 Stuart Street, Kingston, ON K7L3N6, Canada; 2 Sri Lanka Cancer Research Group, Colombo 10230, Sri Lanka; 3 Department of Oncology, Queen’s University, Kingston K7L5P9, Canada; 4 Division of Cancer Care and Epidemiology, Queen’s University Cancer Research Institute, Kingston K7L5P9, Canada; 5 Department of Surgery, Faculty of Medicine, University of Colombo, Colombo 00300, Sri Lanka; 6 National Cancer Institute, Colombo 10230, Sri Lanka; 7 Department of Pharmacology, Faculty of Medicine, University of Colombo, Colombo 00300, Sri Lanka; 8 Health Information Unit, Ministry of Health, Colombo 00300, Sri Lanka

**Keywords:** breast cancer, comorbidities, care delivery

## Abstract

**Purpose:**

Breast cancer is the most common cancer globally as well as in Sri Lanka. Improvements in cancer care have allowed patients to live to an older age. With advancing age, incidence of non-communicable diseases (NCDs) increases. Cancer diagnoses tend to take attention away from the treatment of other comorbidities, given its importance. The objective of this study was to describe healthcare delivery for NCDs among female breast cancer survivors treated at the National Cancer Institute of Sri Lanka (NCISL) and identify opportunities to optimise non-cancer medical care in this cohort.

**Methods:**

A total of 420 women were identified from the breast cancer database at the NCISL, who were 50–80 years at the time of their breast cancer diagnosis, were within 12–24 months from the date of diagnosis, had completed their active cancer treatment and were in complete remission. Of this population, 228 (54%) women who had documented NCDs at the time of diagnosis were identified and were followed-up via telephone to collect details regarding existing comorbidities and the screening and development of new comorbidities.

**Results:**

At the time of cancer diagnosis, 216/228 (95%) of patients had hypertension, 104/228 (46%) had type 2 diabetes and 17/228 (8%) had ischaemic heart disease (IHD). The prevalence of other comorbidities was very low. During the post diagnosis period, 11 patients developed type 2 diabetes, while 2 developed IHD. Osteoporosis screening using dual-energy X-ray absorptiometry scanning was very low at diagnosis 21/228 (9%) but improved in post cancer treatment follow-up 112/228 (49%, p < 0.001). Only 95/228 (42%) were screened for other cancers.

**Conclusions:**

Hypertension was the most prevalent comorbidity while type 2 diabetes and dyslipidaemia were the most common diagnoses post-treatment. In these patients, screening for osteoporosis and other cancers remains very low, emphasising a missed opportunity.

## Introduction

Breast cancer is the most common cancer and the leading cause of cancer death globally with an age standardised incident rate of 46.3 per 100,000 and a mortality rate of 13 per 100 [1]. Breast cancer incidence has been increasing rapidly in Asian, Latin American and African countries [2]. Rising rates of breast cancer in low resource settings are likely related to shifts in population demographics and lifestyle habits including weight gain, sedentary behaviour and alcohol intake [3–5]. In Sri Lanka, breast cancer remains the most common cancer type with an annual age-standardised incidence of 24.3 per 100,000 [6, 7].

The rising incidence and improvements in cancer care have contributed to a growing number of breast cancer survivors, more than half of whom are older than 65 years of age [8]. As these cancer survivors live longer, they are more susceptible to other comorbidities including cardiovascular disease (CVD) due to added risk from complications of treatment and common baselines risk factors [3]. It is important to measure cardiovascular risk factors in the short term, as they can provide an estimate of cardiovascular morbidity and mortality over time [9]. In Sri Lanka, CVD remains the most prevalent cause of morbidity and mortality, and common risk factors such as diabetes, hypertension and obesity have increased [10].

The interaction between cancer and other chronic diseases is complex. There are several common risk factors for CVD and cancer including diet, obesity, physical inactivity, alcohol and reproductive factors [4, 5]. Cancer treatment may lead to early or delayed cardiovascular toxicity that can vary from left ventricular dysfunction, heart failure, coronary ischaemia, hypertension and thromboembolic disease [11, 12]. During a patient’s journey in cancer care, regular follow-up and screening for non-communicable diseases (NCDs) may not be given full attention. The emphasis is typically given to the index cancer diagnosis and may paradoxically lead to inferior health outcomes by not recognising complications from other comorbidities. Furthermore, the point of reference for ongoing care may shift from primary care doctors to oncologists who may have an experience bias towards detection of cancer recurrences rather than screening for and identifying other NCD comorbidities. Therefore, the overall prognosis of many of the breast cancer survivors is dependent on the effective management of their preexisting comorbidities [13]. To the best of authors’ knowledge, no previous study has looked at characterising the healthcare delivery for comorbidities in a cohort of breast cancer patients in Sri Lanka. The objective of this study was to describe healthcare delivery for NCD comorbidities among female breast cancer survivors treated at the National Cancer Institute of Sri Lanka (NCISL) and explore opportunities to optimise their non-cancer medical care.

## Methods

### Setting

The NCISL is the only dedicated cancer hospital in the country. Approximately two-thirds of all cancer patients in Sri Lanka will have some component of care delivered at NCISL. The centre treats approximately 1,200 women with newly diagnosed breast cancer annually. Our team has created an inception cohort of breast cancer patients at the NCISL with ongoing annual follow-up in collaboration with the University of Colombo, Sri Lanka, and Queen’s University in Canada. This inception cohort currently includes over 5,000 breast cancer patients from 2016 to present. Details on cohort creation are described elsewhere [14].

### Eligibility criteria

Patients presenting with newly diagnosed breast cancer who were between 50 and 80 years of age at the time of their breast cancer diagnosis were screened for inclusion in this study. From October to December 2020, patients who had completed a follow-up of 12–24 months prior to 1 October 2020, were recruited into this study. Four- hundred and twenty women were originally identified from the breast cancer database at the NCISL, of which 228 had a diagnosed comorbidity at the start of the study. This age range was selected given the high prevalence of NCDs in this age group. Patients were eligible if: a) they were within 12–24 months from the date of their breast cancer diagnosis; b) have completed their active cancer treatment (surgery, chemotherapy and radiation); c) have no evidence of breast cancer recurrence and d) had documented NCDs at the time of cancer diagnosis (i.e. pre-cancer comorbidity). The comorbidities captured in the registry included diabetes, hypertension, dyslipidaemia, ischaemic heart disease (IHD), heart failure, cerebrovascular disease, chronic kidney disease (CKD), chronic obstructive pulmonary disease (COPD) and liver disease (cirrhosis). These diagnoses were captured during medical assessments by a medical officer and documented in the patient’s medical chart. Relevant lab work that supported these diagnoses was extracted from the charts when available but was not required for study inclusion. These patients were then contacted by phone 12–24 months after their diagnosis to follow-up on the status of their comorbidities. Information on their pre- and post-cancer comorbidities was confirmed and additional details were collected during this phone follow-up. Only comorbidities with a confirmed diagnosis were included for post-diagnosis comorbidities assessment. Other information collected included the medical follow-up available, screening for osteoporosis by a dual-energy X-ray absorptiometry (DXA) scan and screening for any other cancers.

All data were collected by trained abstractors using a standardised questionnaire administered over the phone. Validation of data collection was performed by a board-certified medical specialist observing 20% of these encounters. All patients had consented to participate in the cohort, and this was re-confirmed at the time of the follow-up phone call. If the patient was not able to provide information, an assigned caregiver was approached after obtaining consent from the patient. Patient data were de-identified and stored securely. Only aggregate data are presented in this study. This study was conducted at the NCISL after ethics approval for this study was obtained by the University of Colombo, Sri Lanka.

### Statistical analysis

Descriptive data were reported using counts/percentages and means/standard deviations (SD). Counts before and after treatment were compared using McNemar chi-squared statistic with Yates correction of 0.5 to account for paired sampling, some lower frequency values and upward bias [15]. SPSS statistical software version 20.0 was used to perform calculations [16, 17].

## Results

### Cohort description

The mean (SD) age of our cohort of 228 women was 63.9 (7.2) years, and 21.5% (*n* = 49) were older than 70 years. The majority of the patients (96%) were post-menopausal. The mean (SD) BMI was 26 (4.1) with 61% either being overweight (BMI > 25) or obese (BMI > 30) [18]. There were very low rates of smoking (1%, *n* = 3) and alcohol consumption (1%, *n* = 3) in this cohort. Most patients had Stage II (56%) or Stage III (30%) breast cancer; none had Stage IV disease. Most patients had received adjuvant chemotherapy (64%) and radiation therapy (59%). Two-thirds of patients (75%) were on adjuvant hormonal therapy (Table 1).

### NCD comorbidities

At the time of cancer diagnosis, 104/228 (46%) patients had type 2 diabetes. The majority of patients had hypertension 216/228 (95%), and 17/228 (8%) had IHD. The prevalence of other comorbidities was very low (heart failure (*n* = 1, 0.4%), cerebrovascular disease (*n* = 1, 0.4%), CKD (*n* = 2, 0.8%), COPD (*n* = 1, 1%)) (Table 2).

After a follow-up period of 12–24 months since diagnosis, 11/228 additional patients had a new diagnosis of type 2 diabetes, totalling 115/228 (50%) with type 2 diabetes compared to pretreatment (*p* < 0.001). Also of note, 11 patients were newly diagnosed with dyslipidaemia compared to pretreatment (*p* < 0.01). Only two new patients developed IHD, and one additional patient developed heart failure during this follow-up period, compared to pretreatment (*p* = 0.62, *p* = 0.62, respectively). Other comorbidities including cerebrovascular disease, CKD, liver cirrhosis and COPD had a very low prevalence and did not change significantly post treatment (Table 2).

Osteoporosis screening using DXA scanning was very low at breast cancer diagnosis 21/228 (9%), but a larger proportion were screened at post cancer treatment follow-up 112/228 (49%, *p* < 0.001). However, the majority of patients 116/228 (51%) were still not screened for osteoporosis pre- or post-cancer diagnosis. Most patients on aromatase inhibitors (AI) 74/86 (86%) were screened using the DXA scan. Screening for other cancers also remained low in this cohort; only 95/228 (42%) were screened for other cancers (Table 2).

The majority of these patients were followed up by a government hospital-based clinic outside of the NCISL (98/228 (43%)), or by their primary care provider (80/228 (35%)). Fifty-two patients (23%) saw a specialist in the private sector regarding their comorbidities. Following the completion of their cancer treatment, 14/228 (6%) of patients had follow-up for medical comorbidities through a specialist medical clinic that is now available on site at the NCISL (Table 2). Some patients attended more than one of the above clinics.

## Discussion

To the authors’ knowledge, this is the first study in Sri Lanka which describes the prevalence and care provision of comorbidities among patients with breast cancer. There are several important findings from this study. Firstly, hypertension is the most prevalent comorbidity in our cohort. Secondly, type 2 diabetes and dyslipidaemia are the two most common diagnoses that patients acquire post-treatment among patients with a pre-existing comorbidity at baseline. Thirdly, despite the cardiotoxicity risk of some breast cancer treatments, rates of heart failure and IHD are low. Fourthly, screening rates for common conditions like osteoporosis and other cancers remain very low. Finally, primary care and hospital medical clinics play a pivotal role in healthcare provision for NCD comorbidities. Access to an on-site specialist medical clinic at the cancer centre may further improve management of NCD comorbidity in this patient population.

As expected, the most common comorbidities found in breast cancer patients in Sri Lanka were hypertension and type 2 diabetes. The age-adjusted community prevalence of hypertension in all Sri Lankan adult females is 24% [19], and the prevalence of diabetes in the western province in Sri Lanka, where the NCISL is located, was 19% [20]. Also, the presence of two or more cardiovascular risk factors (including type 2 diabetes, hypertension, dyslipidaemia, obesity) during or after cancer treatment has been found to increase the risk of developing cardiac dysfunction [9]. It is, therefore, prudent to recognise, treat and manage these risk factors efficiently and effectively because of their influence on the overall prognosis [13]. Although our follow-up period was only 12–24 months, the rates of IHD and heart failure were notably low in our study. It is promising to see a low incidence of these comorbidities among these cancer survivors although these numbers may be underestimated due to a lack of active surveillance of IHD or heart failure for non-symptomatic patients. It is also possible that some of these cardiac complications may take longer to manifest. For example, patients with diabetes often have asymptomatic silent ischaemia that goes unrecognised [21]. Given the high rates of cardiovascular risk factors, these patients remain vulnerable to IHD and heart failure [9]. Similarly, the rates of CKD, cirrhosis and COPD were notably low and did not change post treatment.

Our study highlights low rates of screening for osteoporosis and other cancers beyond the index breast cancer diagnosis. Although the majority of patients in this study were postmenopausal, less than 10% of our cohort had been screened for osteoporosis using a DXA scan pre-treatment. This emphasises the need to screen more patients for osteoporosis in this patient group [22].

The majority of patients in our cohort did not undergo screening for other cancers. We would have expected this cohort who regularly attended the primary cancer treatment centre with all resources needed for cancer screening to have had higher incidence of screening tests compared with the general population of a similar age distribution, who do not have ready access to cancer screening services. Breast cancer patients are not only prone to develop recurrence or a synchronous breast cancer but are at an increased risk of developing other cancers. It has been suggested that his may occur because of a genetic predisposition [23] or through interactions with cancer therapies, such as the interaction between endometrial cancer with tamoxifen [24]. The NCISL offers ultrasound scanning of endometrial thickness in patients who are on tamoxifen and also scans for breast cancer recurrence despite no formal protocol. Given that national guidelines for cancer screening are still in development in Sri Lanka, the oncology follow-up clinic is ideally suited to screen for other cancers in these high-risk patients.

This study highlights the pivotal role played by government hospital-based clinics and primary care providers in care provision and managing NCD comorbidities in Sri Lanka. The majority of patients had access to either of these healthcare resources and when needed, sought a specialist. The availability of a specialist medical clinic on site at the NCISL compliments these primary care providers, who often oversaw patients with complex NCD comorbidities. Healthcare seeking in Sri Lanka is often reflexive and symptom based with limited self-initiation of primary prevention [25]. Often there is no referral system that is mandated to see a specialist [26]. Furthermore, the diagnosis of cancer may dilute other primary care follow-up, as it is the more pressing health concern. For these reasons, any healthcare visit including follow-up at the oncology clinic is an opportunity to discuss and implement primary and secondary preventative measures. Acknowledging the high-volumes of patients that are seen in these clinics, we propose the recommendations highlighted in Table 3 based on the Ministry of Health guidelines for managing NCDs [27]. These can easily be incorporated as a preprinted checklist that would make assessments more standardised and time efficient. This has potential to significantly reduce inter-clinician variability without diluting the primary focus of the visit to the cancer clinic. The utility of this checklist can be validated in different settings including a busy outpatient setting like the NCISL.

There are several strengths of this study. We are not aware of any existing literature that describes the common comorbidities and their healthcare delivery among breast cancer patients in Sri Lanka. This was done using a real-world inception cohort with patients presenting to NCISL, which is representative of all breast cancer patients in the country given the wide sociodemographic distribution presenting to this centre. Eligible breast cancer survivors who did not have one or more preexisting comorbidities (*n* = 192) were excluded in this study as our objective was to describe care provision in patient with preexisting comorbidities. We acknowledge that it would be useful to follow patients without comorbidities and we plan to do so in future studies using this longitudinal cohort as a comparator group. Also, future work with longer follow-up time beyond 24 months of diagnosis will capture high-quality important data that may have policy implications. Another limitation of this study is that baseline diagnoses were based on a medical assessment by a medical doctor and were not directly confirmed by investigations potentially leading to misclassification. However, we have made every effort to verify diagnoses using direct communication with the patients which would have minimised the impact of such misclassification. This study is subject to recall bias especially with regard to remote health concerns resulting in potential underestimation in our results.

## Conclusion

This study highlights the interplay of NCDs, especially CVD risk factors with cancer and its treatment. Hypertension was the most prevalent comorbidity in our breast cancer patient cohort, while type 2 diabetes and dyslipidaemia were the most common diagnoses accrued post-treatment. This study recognises the actionable opportunity to scale up the screening and treatment of NCD within a busy cancer clinic. Our findings are generalisable to similar resource setting both regionally and globally.

## Conflicts of interest

The authors state that there are no conflicts of interest.

## Funding

None.

## Figures and Tables

**Table 1.** Cohort details on female breast cancer patients at the NCISL with NCDs.

	Count (*n* = 228)	Percentage^a^
**Demography**		
Age mean (SD) years	64 (7.2)	64 (median)
Post-menopausal	220	97%
Body mass index mean (SD)	26 (4.1)^b^	
Over 25	59	42%
Over 30	27	20%
Current or previous smoking	3	1%
Current or previous alcohol consumption	3	1%
**Details on breast cancer**		
Breast cancer stage at diagnosis		
I	32	14%
II	128	56%
III	68	30%
IV	0	0
Receptor status		
ER/PR positive	161	74%
ER & PR negative	56	26%
ER/PR unknown	(10)	
HER-2 positive	45	21%
HER-2 negative	168	79%
HER-2 unknown	(15)	
Subtype		
Luminal A	126	59%
Luminal B	31	15%
HER-2 amplified	14	7%
Triple negative	42	20%
Unknown	(15)	
Use of neoadjuvant chemotherapy	21	9%
Use of adjuvant chemotherapy	145	64%
Use of adjuvant radiation therapy	135	59%
Use of adjuvant hormonal therapy	170	75%
Tamoxifen	84	37%
AIs	86	38%

a Percentages are based off 228 as the denominator (the total number of included patients in the study)

b Data were available only for 141 patients

ER, Estrogen receptor; PR, Progesterone receptor; HER-2, Human epidermal growth factor receptor 2

**Table 2.** NCDs and care provision in female breast cancer patients at the NCISL.

	At diagnosis		Post treatment		*p* value^b^
	Count (*n* = 228)	Percentage^a^	Count (*n* = 228)	Percentage^a^	
**Comorbidity**					
Diabetes	104	46%	115^c^	50%	<0.001
Hypertension	216	95%	219^c^	96%	0.15
Dyslipidaemia	5	2%	16^c^	7%	<0.01
IHD	17	8%	19^c^	8%	0.29
Heart failure	1	0.4%	2^c^	0.8%	0.62
Cerebrovascular disease	1	0.4%	2^c^	0.8%	0.62
Chronic renal disease	2	0.8%	3^c^	1%	0.62
COPD	3	1%	3^c^	1%	-
Liver disease (cirrhosis)	0	0	4^c^	2%	0.08
**Screening for medical conditions**					
Osteoporosis screening	21	9%	112^c^	49%	<0.001
Osteoporosis screening in patients on AI			74	86%^d^	
Screening for other cancers	8	4%	95^c^	42%	<0.001
**Medical follow-up**					
Hospital clinic (government)	98	43%	85	37%	<0.001
General physician (private)	80	35%	71	31%	<0.01
Medical specialist (private)	52	23%	50	23%	0.54
Medical clinic at NCISL	0	0	14	6%	<0.001

AI, Aromatase inhibitors; COPD, Chronic obstructive pulmonary disease; IHD, Ischaemic heart disease; NCISL, National Cancer Institute of Sri Lanka

a Percentages are based off 228 the total number of patients

b McNemar chi-squared statistic with Yates correction of 0.5 employed

c Denotes cumulative number pre and post treatment

d denominator – 86 patients

**Table 3.** Proposed assessment for NCD in a busy cancer clinic.

At initial assessment
Screen on history Hypertension, diabetes, dyslipidaemia Ischaemic heart disease, heart failure, cerebrovascular disease Renal and liver disease (cirrhosis), COPD, osteoporosis/prior fragility fracture and symptoms of anaemia Personal cancer history Family history of above conditions Medications including over the counter and herbal preparations Allergies Previous surgeries Menstrual and obstetric history Quantify Alcohol, smoking and physical activity Measure Height/weight/BMI Blood pressure Random blood glucose/HbA1C, lipid profile or total cholesterol (non-fasting is appropriate) Full blood count, creatinine and electrolytes Order ONLY if clinically indicated or if part of cancer therapy ECG and echocardiogram Liver profile DXA scan if clinical risk factors/prior fragility fracture
**At follow-up of 3–6 months intervals**
Screen for on history Hypertension, diabetes, dyslipidaemia Ischaemic heart disease, heart failure, cerebrovascular disease Medications and allergies Quantify Alcohol, smoking and physical activity Measure Blood pressure Random blood glucose/HbA1C, lipid profile or total cholesterol (non-fasting is appropriate) Full blood count Order ONLY if clinically indicated or if part of cancer therapy Creatinine and electrolytes Liver profile ECG and echocardiogram Facilitate (NEW at follow-up) Osteoporosis screening (DXA scan) – if not done and at high risk of fractures Age appropriate cancer screening
